# Tensile and Interfacial Loading Characteristics of Boron Nitride-Carbon Nanosheet Reinforced Polymer Nanocomposites

**DOI:** 10.3390/polym11061075

**Published:** 2019-06-21

**Authors:** Venkatesh Vijayaraghavan, Liangchi Zhang

**Affiliations:** Laboratory for Precision and Nano Processing Technologies, School of Mechanical and Manufacturing Engineering, The University of New South Wales, NSW 2052, Australia; liangchi.zhang@unsw.edu.au

**Keywords:** boron nitride–carbon nanosheet, polymer composite, mechanical properties, vacancy defects, molecular dynamics analysis

## Abstract

The discovery of hybrid boron nitride–carbon (BN–C) nanostructures has triggered enormous research interest in the design and fabrication of new generation nanocomposites. The robust design of these nanocomposites for target applications requires their mechanical strength to be characterized with a wide range of factors. This article presents a comprehensive study, with the aid of molecular dynamics analysis, of the tensile loading mechanics of BN–C nanosheet reinforced polyethylene (PE) nanocomposites. It is observed that the geometry and lattice arrangement of the BN–C nanosheet influences the tensile loading characteristics of the nanocomposites. Furthermore, defects in the nanosheet can severely impact the tensile loading resistance, the extent of which is determined by the defect’s location. This study also found that the tensile loading resistance of nanocomposites tends to weaken at elevated temperatures. The interfacial mechanics of the BN–C nanocomposites are also investigated. This analysis revealed a strong dependency with the carbon concentration in the BN–C nanosheet.

## 1. Introduction

The recent advancement in nanotechnology has led to a surge in development of new generation nanocomposites with exceptional properties. Particularly, nanocomposites embedded with 2D nanomaterials such as graphene or boron nitride nanosheets (BNNS), have been extensively researched. The nanocomposite structure consists of a reinforcing nanofiber, embedded by a surrounding matrix–made of a polymer or metallic material. Reinforcement with graphene offers the nanocomposite with better resistance to mechanical loading [1,2,3,4], while BNNS improves thermal stability and oxidation resistance [5,6,7,8]. The distinct properties of these 2D nanostructures have resulted in exploring the material properties of their hybrid offspring, the boron nitride–carbon (BN–C) nanosheet. The laboratory scale fabrication of BN–C nanosheet has been picking up pace with recent improvements in material synthesis processes [9,10]. Analyzing the mechanics of BN–C reinforced nanocomposites is critical for exploiting the functionality of a hybrid nanosheet in designing next generation nanocomposites for a wide range of applications.

The mechanical characteristics of nanocomposites reinforced with conventional nanomaterials have been well studied in the literature. Earlier studies indicate that reinforcing a polymer or metallic sample with even marginal quantities of carbon nanotube (CNT) significantly enhances their mechanical strength [11,12,13,14]. Nanocomposites reinforced with graphene sheets have also been extensively researched [15,16,17,18]. The studies show that the 2D surface of graphene enhances surface contact with the embedding matrix, resulting in a significant enhancement in mechanical properties [19]. Recently, research efforts have been directed towards analyzing the mechanical characteristics of nanocomposites reinforced with BN nanostructures [20,21,22]. In contrast to CNT/graphene, the heterogeneous surface of BN offers an improved load-sharing ability with the surrounding matrix [23]. The thermal stability of BN also makes it more attractive for use in the manufacturing of nanocomposites for protective applications in high thermal or corrosive environments [24,25]. Buoyed by the contrasting properties of graphene and BNNS, there is a clear need to investigate the mechanical characteristics of nanocomposites reinforced with hybrid BN–C nanostructures. Indeed, some computational studies have confirmed the attractive physical qualities of stand-alone BN–C nanostructures in response to mechanical and thermal loads [26,27].

The mechanical properties of a nanocomposite can be significantly affected by variations in the material type, the defects, and the geometry of the reinforcing nanostructure. A limited number of studies examine the effect of a defective nanostructure on the mechanical properties of nanocomposites [28,29]. A comprehensive study that thoroughly investigates the effect of various factors on the mechanical properties of nanocomposites is clearly lacking in the literature.

The knowledge gaps outlined above show that the mechanical properties of nanocomposites reinforced with BN–C nanosheets must be characterized based on a wide range of underlying factors, such that their engineering application can be possible. Hence, the objective of the present study is to provide a comprehensive analysis of the tensile loading behavior of nanocomposites by systematically varying the nanosheet configuration, defects, and operating temperature. The interfacial mechanics of the nanocomposite structure reinforced with BN–C nanosheets of varying carbon concentration will also be studied. It is anticipated that the findings will provide useful design inputs for making a new generation of nanocomposites suitable for mechanical and structural loading applications.

## 2. Computational Model

**Figure 1:**
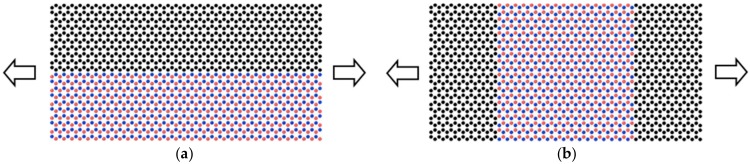
Types of hybrid Boron Nitride-Carbon nanosheets considered in the study. The graphene and BN segments can be arranged in (**a**) parallel or in (**b**) series with respect to the axial direction. The atoms depicted in the ochre color represent boron, those in the blue color represent nitrogen, and those in the black color represent carbon.

The computational study described in this paper is entirely modelled using the classical molecular dynamics (MD) simulation approach. The reinforcing nanofiber is a BN–C nanosheet, which is formed by varying the percentage of carbon atoms in the BNNS, defined as the ratio of carbon atoms to the total of the boron and nitrogen atoms. Two types of BN–C nanosheet are considered in the study based on the lattice structure, viz., series and parallel BN–C nanosheets (as shown in Figure 1). The matrix structure consists of polyethylene molecules (PE) consisting of 20 repeat units of a–CH_2_–monomer. The simple structure of the PE molecule effectively reduces the computational cost while acting as a representative system for all polymer nanocomposites, as in previous studies [23,30]. As such, the nanocomposite structure will consist of three parts: (1) a BN–C nanosheet; (2) a matrix made of PE molecules; and (3) the interface formed between the nanosheet and the matrix. The interaction between the carbon and hydrogen atoms in the PE chain is modelled using the Modified Embedded Atom Method (MEAM) potential for hydrocarbons [31]. The MEAM potential is particularly effective for modelling the mechanical behavior of polymeric hydrocarbons [32,33]. For the case of BN–C nanosheets, the interactions between B–N, B–C, and N–C atoms is described using modified Tersoff potential [34], with the interaction parameters taken from [35], and the interactions between C–C atoms are modelled using the Brenner’s second generation Reactive Bond Order (REBO) potential function [36]. Combining the modified Tersoff potential with REBO for modelling BN–C nanosheets enables an accurate description of the mechanics of BN–C nanosheets comparable with the computationally expensive ab initio, or density functional theory (DFT), technique [37]. The non-bonded interactions between the atoms of the PE chains and the nanosheet are described using the Lennard–Jones (LJ) potential, with parameters extracted from [38,39,40].

**Figure 2:**
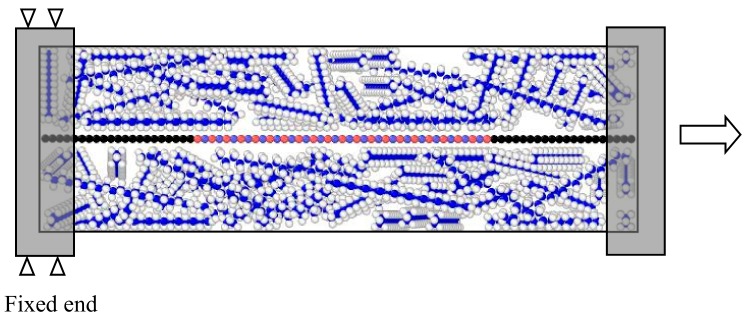
Description of tensile loading of the BN–C nanosheet reinforced PE nanocomposite in the study. In the PE matrix, carbon atoms are depicted by blue colored balls and hydrogen atoms are depicted by white colored balls. In the BN–C nanosheet, the carbon, boron, and nitrogen atoms are depicted by black, ochre, and purple colored balls, respectively. The figure is a representative image of the simulation box with enhanced distinction between the matrix and nanosheet regions for easier visualization.

The work described in this paper focusses on the tensile loading characteristics and interfacial mechanics of PE nanocomposites. At first, a BN–C nanosheet with a specific concentration of carbon atoms was placed at the geometric center of a simulation box, with dimensions 50 Å × 50 Å × 70 Å. The remaining space in the simulation box was then filled with 150 PE molecules randomly using the Packmol software [41]. At first, the system was subjected to equilibration under conjugate–gradient minimization. After this, the system was equilibrated under an NVE ensemble for 30 ps. This was, again, followed by an NVT equilibration, incorporating a Langevin thermostat at 0 K for 30 ps. These subsequent equilibration processes resulted in a nanocomposite structure with evenly distributed PE molecules surrounding the core nanosheet at the center, which could then be subjected to tensile loading. Before the application of mechanical load, the NVT ensemble incorporating Langevin thermostat was used to equilibrate the system by gradually ramping the temperature from 0 K to the desired temperature. The tensile loading was modelled by fixing the two ends of the nanocomposite structure after it was equilibrated to the desired temperature. The tensile mechanics of the nanocomposite were tested at three temperatures: 100 K, 200 K, and 300 K. As shown in Figure 2, the atoms at one end were constrained, while the atoms at the other end were subjected to constant displacement to model the tensile loading. At the end of each fixed displacement, the system was equilibrated for 1 ps and the displacement was carried out until the nanocomposite fractured. All the simulations described in this paper were carried out on the LAMMPS open source software [42], which is an acronym for the large scale atomic/molecular massively parallel simulator package.

## 3. Tensile Loading Characteristics of Graphene and BNNS Reinforced Nanocomposites

**Figure 3:**
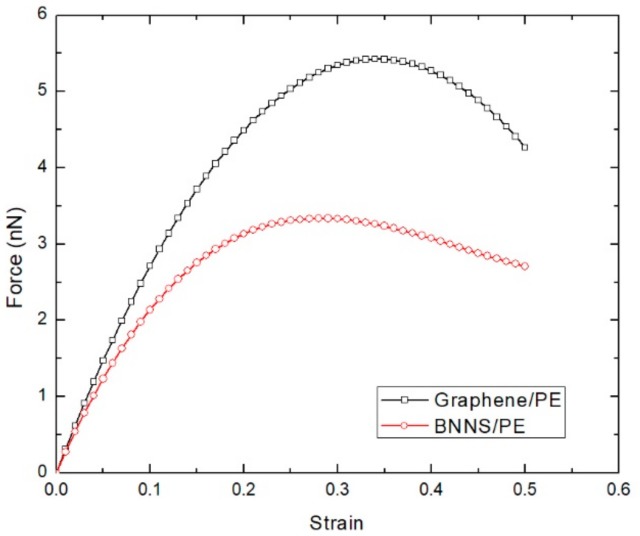
Force–strain plot of the graphene/PE and boron nitride nanosheet/PE nanocomposites at 300 K.

**Figure 4:**
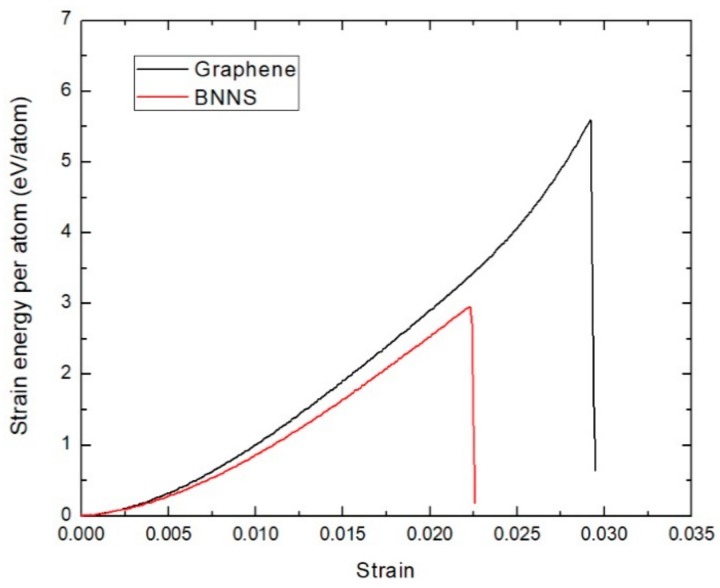
Force–strain plot of graphene and boron nitride nanosheet components in the PE nanocomposites at 300 K.

The tensile loading characteristics of graphene and BNNS reinforced nanocomposites at 300 K are investigated first. The dimension of graphene sheet is 19.89 Å × 68.92 Å and for BNNS is 20.58 Å × 67.47 Å. These dimensions ensure the geometry is standardized for these two distinct sheets embedded in the PE matrix. The force–strain plots of the graphene and BNNS reinforced nanocomposite under tensile loading is shown in Figure 3. Regardless of the sheet type, the tensile force increases linearly with strain (*ε*), for low strain values. At higher strain values, the curve becomes more parabolic with the tensile force reaching a peak value. When the tensile loading is further increased, the tensile force gradually decreases. The obtained force–strain characteristics of the graphene/PE and BNNS/PE nanocomposites from our simulation match the trend normally observed in PE variants undergoing tensile loading [43]. It can also be seen that the graphene/PE nanocomposite shows superior performance under tensile loading compared to that of BNNS/PE nanocomposite. The variation in strain energy per atom of the embedded graphene and BNNS nanosheet component is depicted in Figure 4. The strain energy of the graphene is consistently higher than that of the BNNS component. The maximum strain energy indicates the measure of energy required to initiate the fracture of the embedded nanosheet component. The maximum strain energy threshold exhibited by the graphene is much higher than that of the BNNS, which results in enhanced tensile loading performance of the graphene/PE nanocomposite.

**Figure 5:**
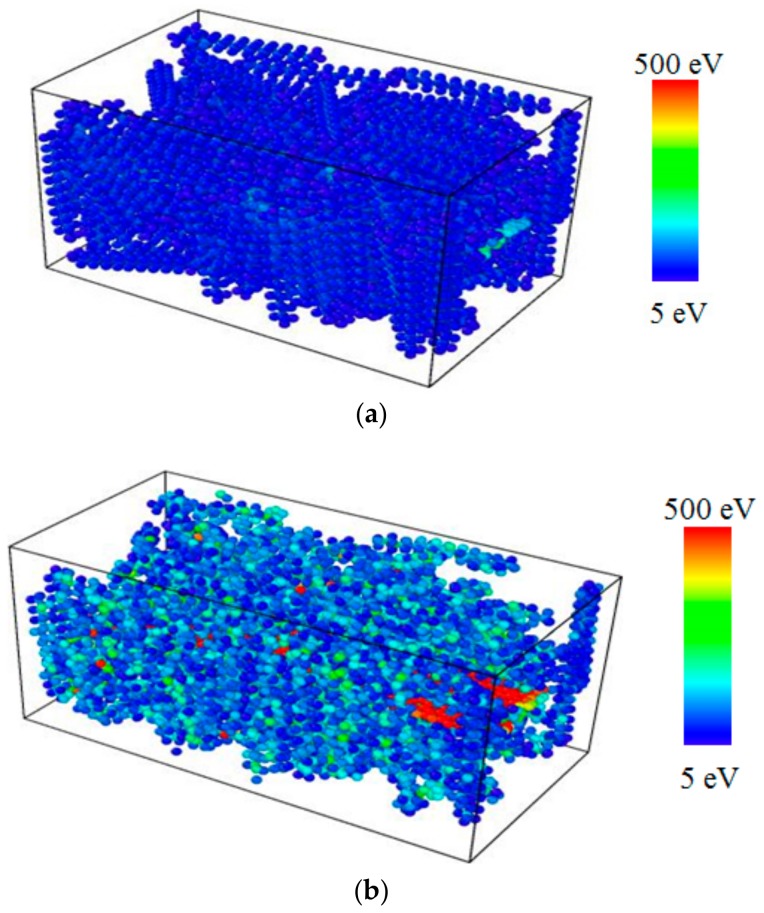
Computer generated screen shots of the graphene/PE nanocomposite under tensile loading when (**a**) *ε* = 0.0 and (**b**) *ε* = 0.34.

The computer-generated screen shots of the graphene reinforced PE nanocomposite are illustrated in Figure 5. Before the application of tensile loading, the PE nanocomposite maintained a stable equilibrated structure. The PE molecules formed a consolidated mass surrounding the embedded graphene sheet. Application of tensile loading resulted in stretching of the PE nanocomposite structure, which disintegrated and fragmented the graphene/PE nanocomposite structure.

## 4. Tensile Loading Characteristics of BN–C/PE Nanocomposites

### 4.1. Effect of Chirality and Lattice Structure of BN–C Nanosheet

**Figure 6:**
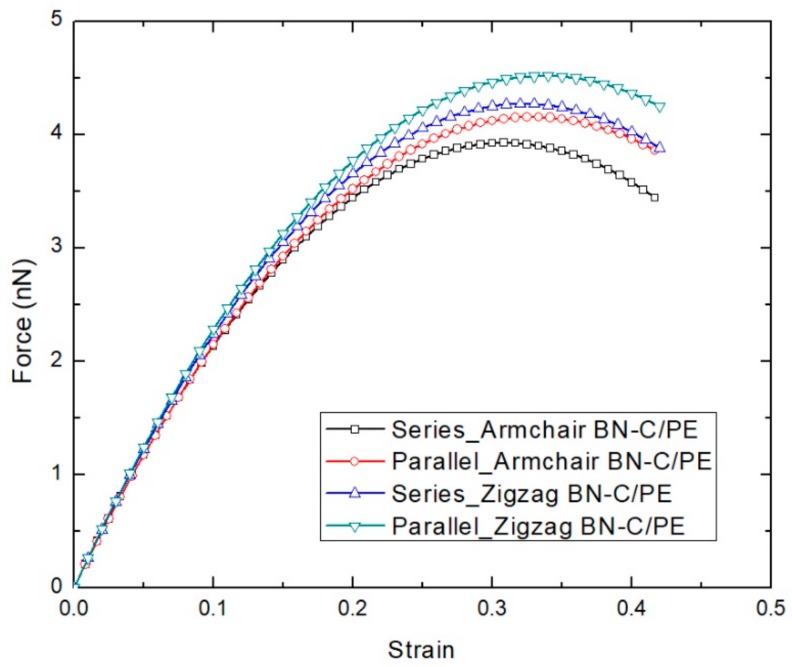
Force–strain plot of BN–C/PE nanocomposite variants at 300 K.

**Figure 7:**
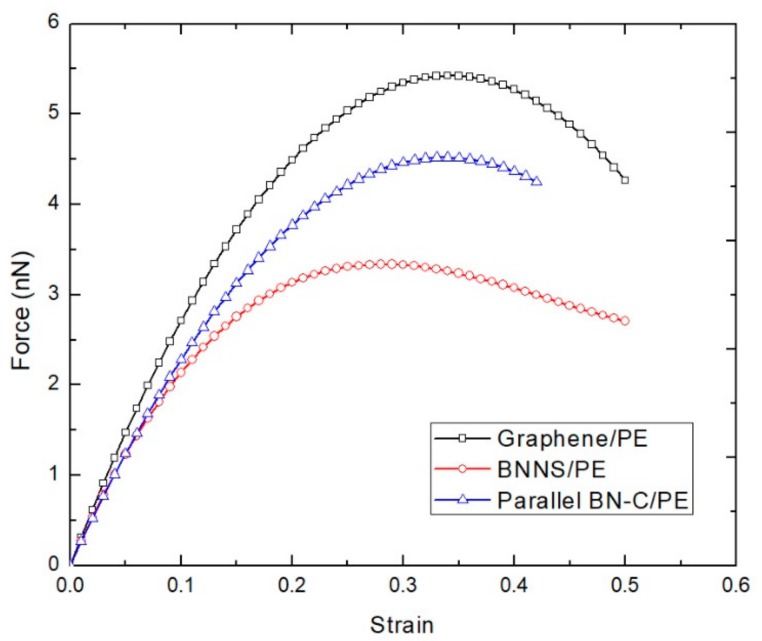
Force–strain plot of BN–C/PE nanocomposite variants at 300 K.

The effect of chirality and the lattice structure of the BN–C nanosheet on the tensile loading mechanics of BN–C/PE nanocomposites are investigated first. BN–C nanosheets of armchair and zigzag orientations with a carbon concentration of 50% are considered. Table 1 lists the specifications of the armchair and zigzag BN–C nanosheets deployed in this work. The atomic structures in the armchair and zigzag BN–C nanosheets are further rearranged to form a series and parallel lattice structures, resulting in a total of 4 varying BN–C nanosheet configurations. The tensile loading characteristics of all the variants of the BN–C/PE nanocomposites are shown in Figure 6. Regardless of the orientation, the zigzag BN–C/PE nanocomposite shows improved resistance to tensile loading compared to that of its armchair counterpart. Further, regardless of the orientation, the BN–C/PE nanocomposite with a parallel BN–C nanosheet shows slightly improved performance compared to that of its series counterpart. Based on the discussion from Section 3, the graphene nanosheet exhibits higher strength compared to a BNNS when loaded under tension. Hence, the orientation of a graphene sheet that is aligned parallel to a weaker BNNS forming a parallel BN–C nanosheet improves its tensile resistance, compared to the series BN–C nanosheet. This observation is also confirmed by previous studies on the tensile loading mechanics of pristine BN–C nanotubes by Zhang et al. [26]. For comparison, Figure 7 compares the tensile loading curves of PE nanocomposite reinforced with graphene, BNNS, and a parallel BN–C nanosheet. The performance of the BN–C/PE nanocomposite falls within the tensile loading curves of graphene and BNNS reinforced PE nanocomposites. Based on a previous study conducted by the authors [44], the Young’s modulus of a BN–C nanosheet is always within the limits of the Young’s modulus of graphene and BNNS. These variations in tensile strength offered by the unique structure of the BN–C nanosheet could provide a useful guideline for the manufacturing of BN–C/PE nanocomposites for high strength applications.

### 4.2. Effect of Vacancy Defects in BN–C Nanosheet Lattice

**Figure 8:**
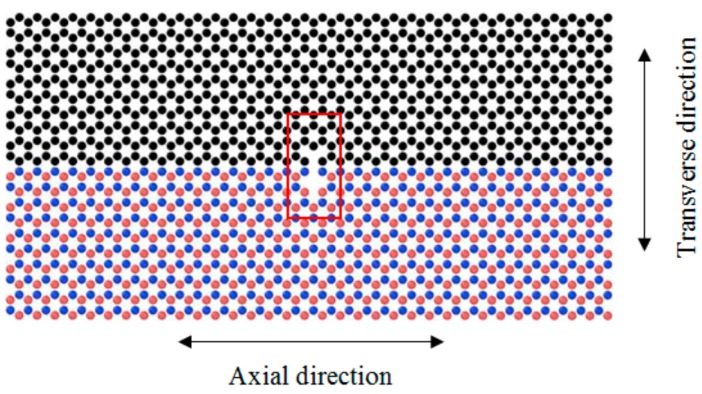
The vacancy defects are constructed by removing each of the B, N, and C atoms enclosed inside the red colored rectangle. The defects are then expanded along the axial or transverse direction relative to the nanosheet loading direction.

**Figure 9:**
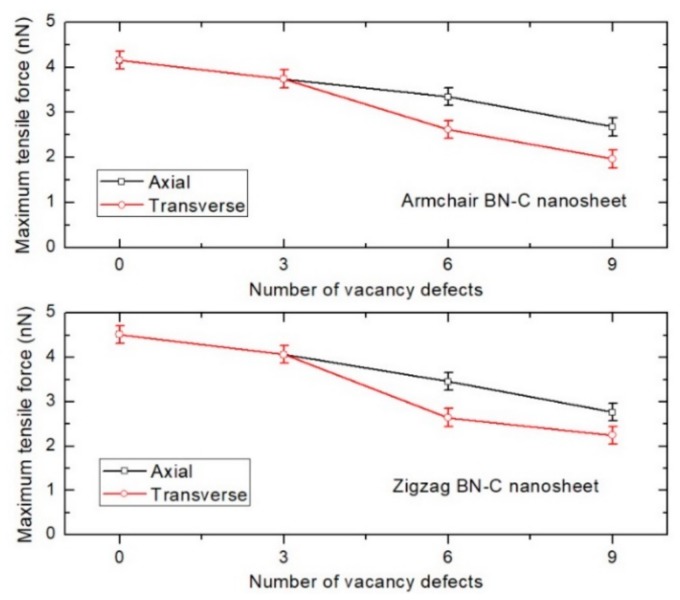
Plot of the maximum tensile force of BN–C/PE nanocomposites with varying vacancy defect concentrations at 300 K.

The effect of a vacancy defect on the lattice structure of the BN–C nanosheet in the tensile loading characteristics of the BN–C/PE nanocomposite is investigated next. For this study, the parallel BN–C nanosheet in a zigzag orientation, consisting of 50% of carbon atoms, is considered as the reinforcement. The vacancy defects are constructed in the nanosheet lattice by removing 3 atoms (B, N, and C), simultaneously resulting in a defected nanosheet. Axial and transverse defect expansion is considered to further include the effect of the defect location in the nanosheet (Figure 8). The variation of maximum tensile force of the various BN–C/PE nanocomposites with defected BN–C nanosheet reinforcement is depicted in Figure 9. The presence of vacancy defects in the reinforcing nanofiber can adversely affect the mechanical properties of the BN–C/PE nanocomposites. It can further be noted that while an increase in defect concentration itself can reduce the tensile force, the extent of reduction is strongly dictated by the location of the vacancy defects. For instance, defects across the transverse direction of the BN–C nanosheet rapidly decreases the tensile resistance of the nanocomposite compared to that of the defects located across the axial direction. This phenomenon is obvious due to the reason that the vacant sites in a direction perpendicular to the loading direction facilitate a rapid fracture of the nanosheet under tensile loading. This observation provides an important guideline for manufacturing nanocomposites in that care must be taken to ensure that the transverse defects in the reinforcing nanofiber be minimized to avoid a rapid decrease in the strength of the nanocomposite.

### 4.3. Effect of Temperature

**Figure 10:**
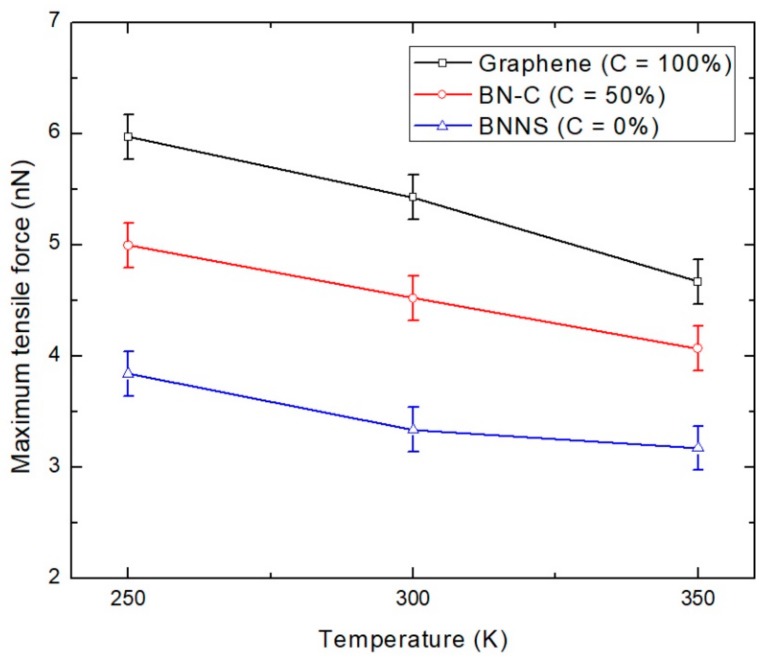
Tensile loading characteristics of the BN–C/PE nanocomposites at various temperatures. The carbon concentration of the nanosheet reinforcement is described in the figure legend.

The effect of temperature on the tensile loading characteristics of BN–C/PE nanocomposites is also investigated. The tensile loading is carried out at 250 K, 300 K, and 350 K, considering the operating temperature range of the BN–C/PE nanocomposites. The variation of the tensile force of the BN–C/PE nanocomposite with varying carbon concentration at various temperatures is shown in Figure 10. The resistance to tensile loading decreases at elevated temperatures, which occur as a result of an increase in the associated thermal stress of atoms at high temperatures. The percentage reduction in the maximum tensile force of the BN–C/PE nanocomposites from transiting from 250 K to 350 K is presented in Table 2. The reduction in tensile loading is less pronounced for a BNNS/PE nanocomposite compared to its other two counterparts. This observation is similar to the previous studies in [45,46], which found that the BNNS exhibits much better thermal resistance than graphene. This provides an interesting observation that the BN–C nanosheet offers an optimal mix of a superior tensile loading performance and a better thermal resistance for the BN–C/PE nanocomposite.

## 5. Interfacial Mechanics of BN–C/PE Nanocomposites

**Figure 11:**
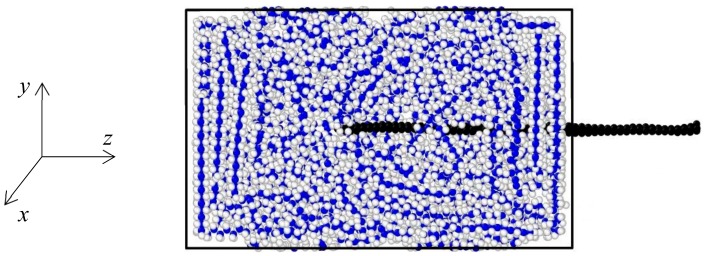
Pull-out mechanics of the graphene sheet (BN–C nanosheet with 100% carbon atoms) from the BN–C/PE nanocomposite at 300 K.

This section presents a study on the interfacial mechanics of BN–C/PE nanocomposites. The interfacial strength of the BN–C/PE nanocomposites is determined through a pull-out test of the BN–C nanofiber, at varying carbon concentrations conducted at 300 K. The equilibrated structure of the BN–C/PE nanocomposite is obtained by following the procedure as described in Section 2. The two ends of the PE matrix are then constrained to rigidly secure the matrix during the nanosheet pull-out process. Periodic boundary conditions are applied in the *x* and *y* directions. This is followed by fixing the atoms at either end of the nanosheet to avoid any relative displacement between them. After this, the reinforcing nanosheet is gradually pulled out of the matrix by applying a constant outward displacement to atoms at one end of the nanosheet, as shown in Figure 11. Three different BN–C sheet configurations are considered based on the carbon atom concentrations of 0% (BNNS), 50% (BN–C), and 100% (graphene).

**Figure 12:**
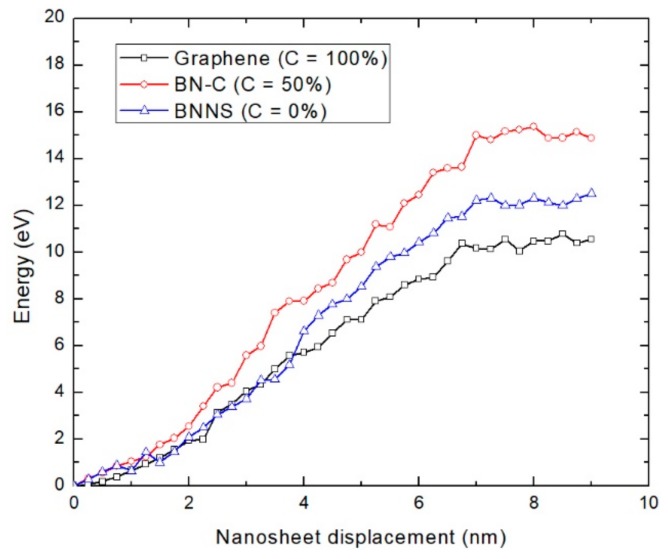
Plot of the variation of energy during the pull-out process of BN–C nanosheets with varying carbon atoms from the PE matrix at 300 K.

The variation in potential energy (*E_pull-out_*) between the matrix and the nanosheet while pulling out the nanosheet from the PE matrix for three various BN–C nanosheet configurations is illustrated in Figure 12. The plot shows that the potential energy gradually increases when the nanosheet is under displacement within the matrix zone. After this process, the potential energy reaches a steady threshold value with no significant variation, as the nanosheet is already separated from the matrix. It can be noted from this plot that the potential energy required to pull-out the nanosheet is strongly influenced by the element configuration in the BN–C nanosheet. This observation is consistent with the interfacial mechanics of the pull-out process of a BN–C nanotube from a PE matrix, as reported by Zhang et al. [23]. The detailed investigations conducted by Zhang et al. showed that the interfacial strength of the nanosheet, with its embedding matrix, is strongly dictated by its surface quality. The graphene sheet, being a homogenous nanostructure, has an almost smooth surface, which facilitates easier removal and results in lower pull-out energy. The presence of dissimilar atoms in the BNNS and BN–C nanosheet increases the surface roughness, which could have contributed to the resulting higher energy required to separate the nanosheet from the PE matrix.

The interfacial shear strength (ISS), *τ* offered by the nanosheet is a critical mechanical property that determines the load-transfer ability of the nanocomposite [47,48]. The energy required to pull-out the nanosheet (*E_pull-out_*) from the matrix can be expressed as the product of *τ* with the surface area of the nanosheet in contact with the matrix. In this study, *E_pull-out_* is represented by the final steady-state values of the potential energy, as can be seen in Figure 12. Hence, during the course of pull-out process, *τ* can be computed from *E_pull-out_*, as defined by the following equation [23],

(1) $$E_{pull-out}=\int_{0}^{L}W\tau \left(L-\delta \right)d\delta$$

where *L* and *W* represents the length and width of the nanosheet, respectively, and *δ* denotes the incremental displacement of the nanosheet from the nanocomposite matrix. Equation (1) is rearranged to obtain ISS as follows:

(2) $$\tau =\frac{E_{pull-out}}{WL^{2}}$$

**Figure 13:**
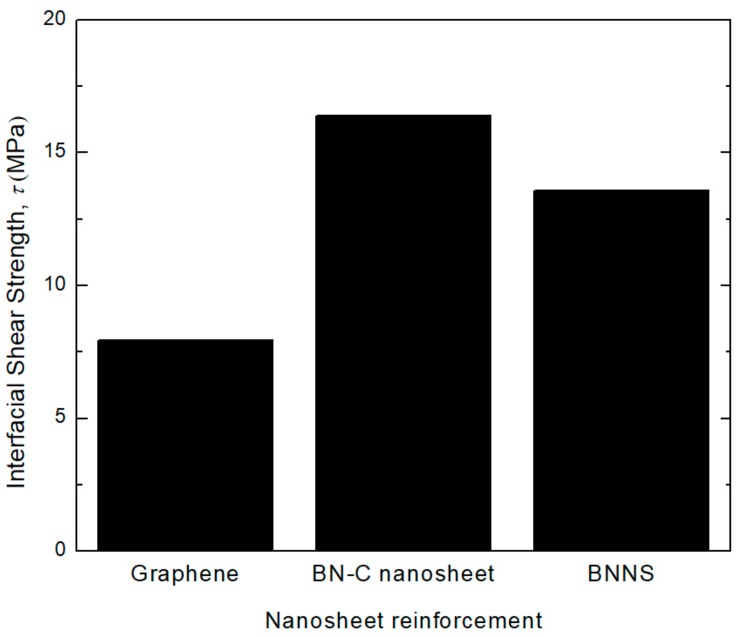
Interfacial shear strength of the nanosheet type reinforcement on the PE matrix.

The computed ISS, *τ* of nanocomposites with three varying nanosheet configurations is depicted in Figure 13. As the *E_pull-out_* of the BN–C nanosheet is much higher compared to the other counterparts, the resulting ISS is also much higher. This analysis shows that embedding the PE matrix with a BN–C nanosheet improves the load sharing ability of the nanocomposite. Combined with the observations drawn from Section 4.3, this study presents an interesting potential for BN–C nanosheets to be used in the manufacture of high strength and temperature resistant durable nanocomposites.

## 6. Conclusions

This study carried out a comprehensive investigation into the tensile loading characteristics of BN–C reinforced PE nanocomposites. The tensile load-carrying capacity of the BN–C nanocomposites is influenced by the lattice structure and geometry of the reinforcing BN–C nanosheet. The presence of vacancy defects in a BN–C nanosheet also negatively affects the nanocomposite’s resistance to tensile loading, dictated strongly by the defect location. The analysis of the sensitivity of tensile force to loading temperature revealed that the sensitivity seems to be slightly less pronounced for a BN–C nanosheet with reduced carbon concentration. The interfacial load-sharing characteristics of the BN–C nanocomposite show that the hybrid surface of a BN–C nanosheet provides superior interfacial contact with the embedding matrix. These findings are valuable for the design and manufacturing of BN–C reinforced PE nanocomposites for a range of engineering applications.

## Figures and Tables

**Table 1:** Specifications of the BN–C nanosheet reinforcement.

Loading Direction	BN–C Dimensions (*W* × *L*)	Total Number of Atoms
Armchair	19.09 Å × 66.89 Å	496
Zigzag	20.58 Å × 67.47 Å	540

**Table 2:** Percentage reduction of the maximum tensile force of the BN–C nanosheet with defects when the temperature is increased from 300 K to 900 K.

Sheet Type	Reduction of Maximum Tensile Force (%)
Graphene	21.81
BN–C nanosheet	18.55
BN nanosheet	17.39
